# Supplementary material for: An Isoform of the Eukaryotic Translation Elongation Factor 1A (eEF1a) Acts as a Pro-Viral Factor Required for Tomato Spotted Wilt Virus Disease in Nicotiana benthamiana
Source: Viruses. 2021 Oct 30;13(11):2190. doi: 10.3390/v13112190 (PMC8619209; doi:10.3390/v13112190)
Supplement: Supplementary file 1 [file viruses-13-02190-s001.zip › Table S4 Putative plant host factors selected for functional validation in N. benthamiana.pdf]

Protein ID	Functional gene discription/ annotation	significant in RNP plant fraction	yeast/plant overlap	Tested in VIGS	Effect on TSWV disease development
NbS00023178g0001.1	Eurkaryotic Elongation factor 1-alpha			+	+
NbS00008317g0004.1	Charged multivesicular body protein 1			+	+
NbS00034922g0015.1	Beta(1,3)-glucanase regulator			+	+
NbS00056501g0001.1	Ammonium transporter			+	+
NbS00062251g0003.1	Sugar transporter protein 13	+	+	+	
NbS00058697g0004.1	Hexose transporter	+	+	+	
NbS00005708g0012.1	DNA-J homolog 13	+	+	+	
NbS00037522g0005.1	Putative ATP-dependent RNA helicase YLR419W		+	+	
NbS00023311g0013.1	Monosaccharid transporter		+	+	
NbS00019858g0012.1	Long-chain-fatty-acid--CoA ligase 1		+	+	
NbS00023331g0003.1	Heat shock protein SSA1		+	+	
NbS00040361g0003.1	Heat shock protein 70		+	+	
NbS00036611g0008.1	Eukaryotic translation initiation factor 2 subunit gamma		+	+	
NbS00039262g0004.1	ER-retained PMA1-suppressing protein 1		+	+	
NbS00009889g0007.1	Endoplasmic reticulum transmembrane protein 1		+	+	
NbS00006769g0021.1	DnaJ-like protein		+	+	
NbS00015581g0008.1	DnaJ-like chaperone JEM1		+	+	
NbS00027624g0003.1	Calcium-transporting ATPase 2		+	+	
NbS00011481g0021.1	ATP-dependent molecular chaperone HSC82		+	+	
NbS00054171g0002.1	SSUH2 homolog isoform x2, HSP40-like	+		+	
NbS00033465g0001.1	Probable mitochondrial chaperone BCS1-B	+		+	
NbS00010523g0001.1	Pleiotropic drug resistance protein 1	+		+	
NbS00061216g0001.1	Pathogenesis-related protein 1	+		+	
NbS00023594g0004.1	Inorganic phosphate transporter	+		+	
NbS00006593g0102.1	Harpin inducing prot 1-like 18	+		+	
NbS00010129g0001.1	Glucan endo-1,3-beta-glucosidase	+		+	
NbS00002670g0023.1	Cytochrome b561	+		+	
NbS00022441g0017.1	Cysteine-rich receptor-like protein kinase 2	+		+	
NbS00013764g0007.1	Catalase isozyme 1	+		+	
NbS00001150g0003.1	Calcium-dependent lipid-binding (CaLB domain) family protein	+		+	
NbS00020002g0005.1	ABC transporter B family member 21	+		+	
NbS00006199g0011.1	Inorganic phosphate transporter	+		+	
NbS00003054g0022.1	Embryo-specific protein ATS3B	+		+	
NbS00057576g0008.1	Clathrin light chain 2	+		+	
NbS00056699g0001.1	V-type proton atpase subunit f			+	
NbS00014830g0004.1	V-type proton ATPase subunit E			+	
NbS00027173g0010.1	Vacuolar protein sorting-associated protein			+	
NbS00009194g0006.1	Ubiquitin-associated (UBA)/TS-N domain-containing protein			+	
NbS00014326g0003.1	Transmembrane protein 205 (likely LEA/chaperone)			+	
NbS00027158g0003.1	Syntaxin-related protein			+	
NbS00041675g0001.1	Subtilisin-like protease			+	

NbS00024294g0008.1	Ribosomal protein S24			+	
NbS00055226g0005.1	Ribosomal protein S19			+	
NbS00006294g0017.1	Respiratory burst oxidase homolog protein			+	
NbS00020069g0017.1	Putative ferredoxin-dependent glutamate synthase 1			+	
NbS00038999g0004.1	Pleiotropic drug resistance protein 1			+	
NbS00006080g0002.1	Monosaccharid transporter			+	
NbS00000522g0011.1	Mitochondrial inner membrane protein OXA1-like			+	
NbS00056412g0006.1	Metaxin, mitochondrial outer membrane import complex			+	
NbS00014166g0101.1	Hypothetical protein			+	
NbS00003404g0002.1	Hydroxyproline-rich glycoprotein family			+	
NbS00021573g0008.1	Hydroxyproline-rich glycoprotein			+	
NbS00013845g0026.1	HSP80-like			+	
NbS00020204g0009.1	EDR4-like (ENHANCED DISEASE RESISTANCE4)			+	
NbS00001000g0012.1	DNAJ homolog			+	
NbS00040506g0007.1	Cysteine protease			+	
NbS00035854g0013.1	Charged multivesicular body protein 2a			+	
NbS00027166g0001.1	Aspartic protease			+	
NbS00004038g0017.1	50S ribosomal protein L1-like			+	
NbS00032958g0009.1	2-Oxoglutarate dehydrogenase			+	
NbS00021840g0003.1	26S protease regulatory subunit 6A			+	

Table S4. Putative plant host factors selected for functional validation in *N. benthamiana*. Combined list of host factors identified as significant in RNP containing fraction of *N. benthamiana* and the overlap in functional annotations of proteins present in plant and yeast.
