# Supplementary material for: An Isoform of the Eukaryotic Translation Elongation Factor 1A (eEF1a) Acts as a Pro-Viral Factor Required for Tomato Spotted Wilt Virus Disease in Nicotiana benthamiana
Source: Viruses. 2021 Oct 30;13(11):2190. doi: 10.3390/v13112190 (PMC8619209; doi:10.3390/v13112190)
Supplement: Supplementary file 1 [file viruses-13-02190-s001.zip › Table S5 Predicted small RNAs formed by the TRVeEF1A-silencing constructs.pdf]

**Silencing construct**	***N. benthamiana* Genes targeted for VIGS**	**predicted sRNAs**
TRV::*eEF1A.A1*	Niben101Scf08618g01001.1	367
	Niben101Scf08618g01002.1	13
TRV::*eEF1A.A2*	Niben101Scf08618g01002.1	377
	Niben101Scf08618g01012.1	377
	Niben101Scf08618g00005.1	356
	Niben101Scf08618g01001.1	240
	Niben101Scf34389g00002.1	153
	Niben101Scf12941g01003.1	111
TRV::*eEF1A.B*	Niben101Scf07423g04011.1	239
	Niben101Scf04639g06007.1	44
	Niben101Scf12941g01003.1	2

Table S5. Targets of the three *eEF1A* VIGS constructs in *N. benthamiana.* The *N. benthamiana* eEF1A isoforms that are targeted by the three different VIGS constructs, TRV::*eEF1A.A1,* TRV::*eEF1A.A2* and TRV::*eEF1A.B* are indicated*.* The number of predicted small RNAs (sRNAs), derived from the VIGS constructs, per individual *eEF1A* isoform is indicated.
