# Supplementary figures and images for: An Isoform of the Eukaryotic Translation Elongation Factor 1A (eEF1a) Acts as a Pro-Viral Factor Required for Tomato Spotted Wilt Virus Disease in Nicotiana benthamiana
Source: Viruses. 2021 Oct 30;13(11):2190. doi: 10.3390/v13112190 (PMC8619209; doi:10.3390/v13112190)

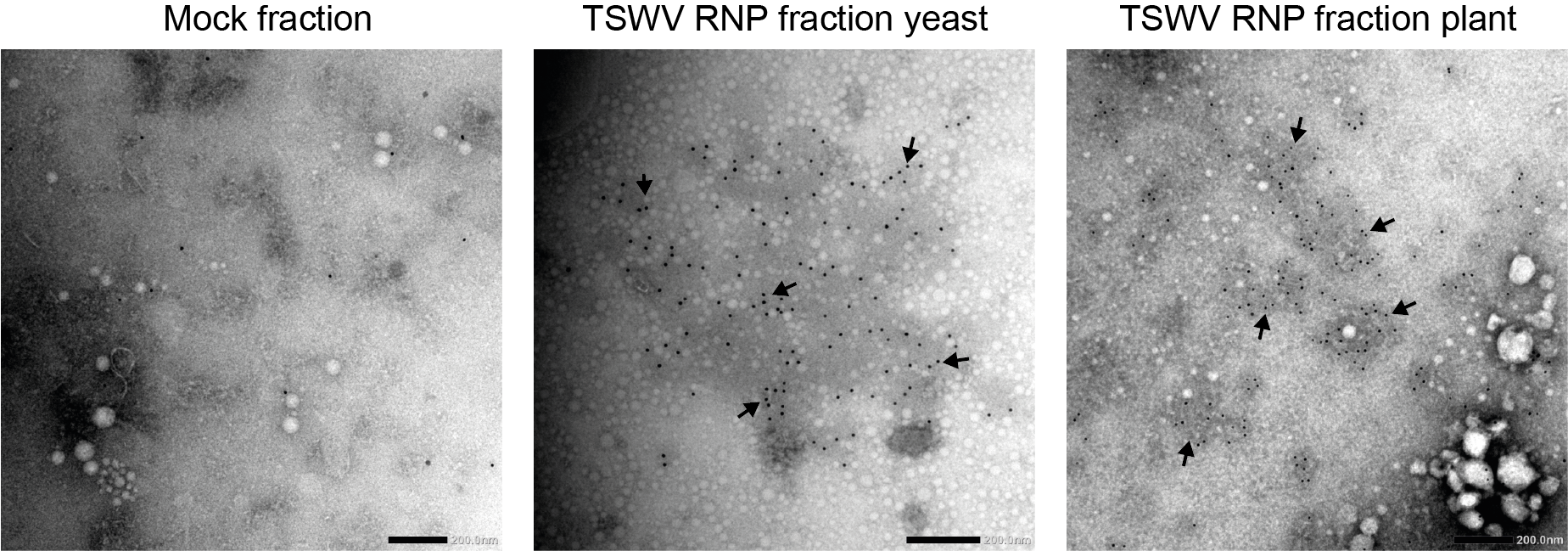

Supplement: Supplementary file 1 [file viruses-13-02190-s001.zip › Figure S1 EM of RNPs.tif]

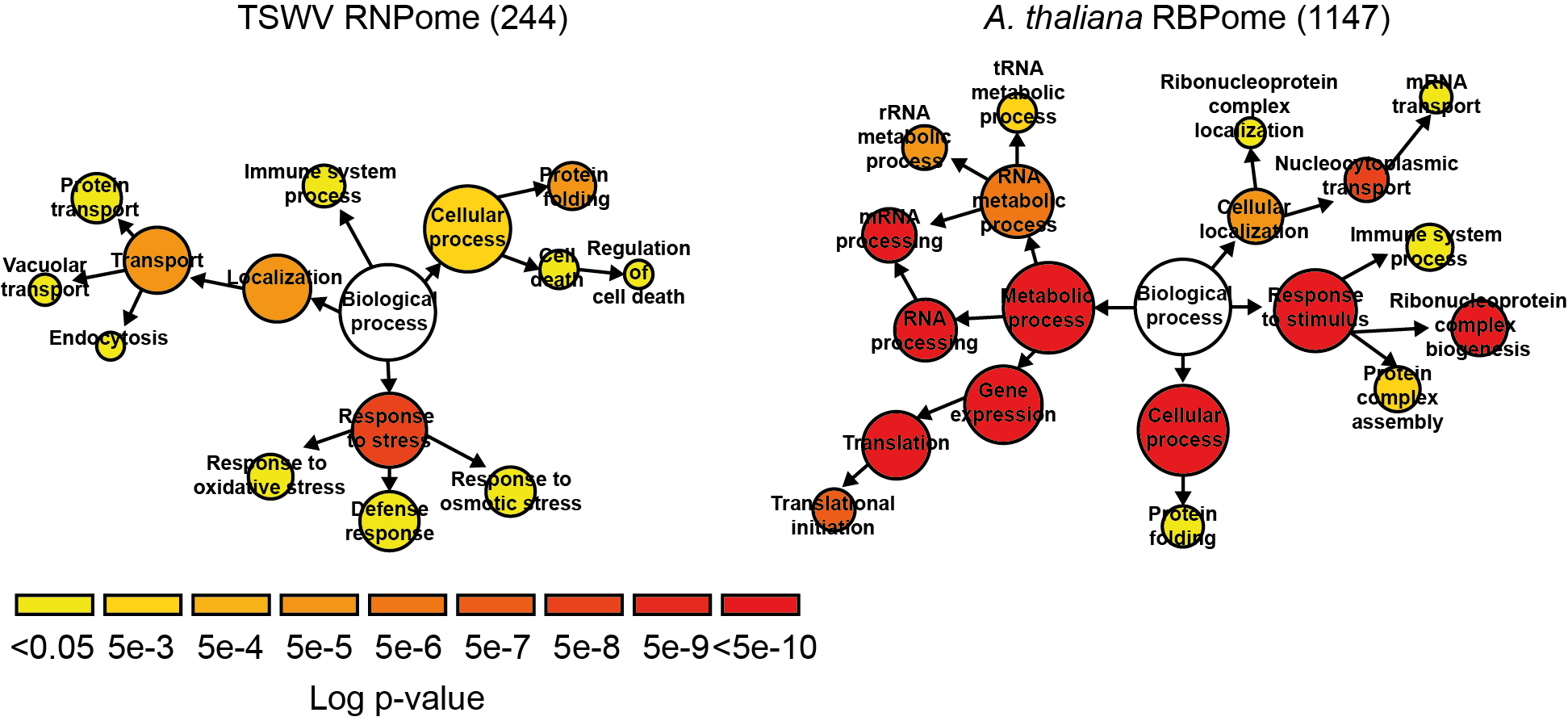

Supplement: Supplementary file 1 [file viruses-13-02190-s001.zip › Figure S2 GO comparison between the Arabidopsis RBPome and TSWV RNPome-RGB.tif]

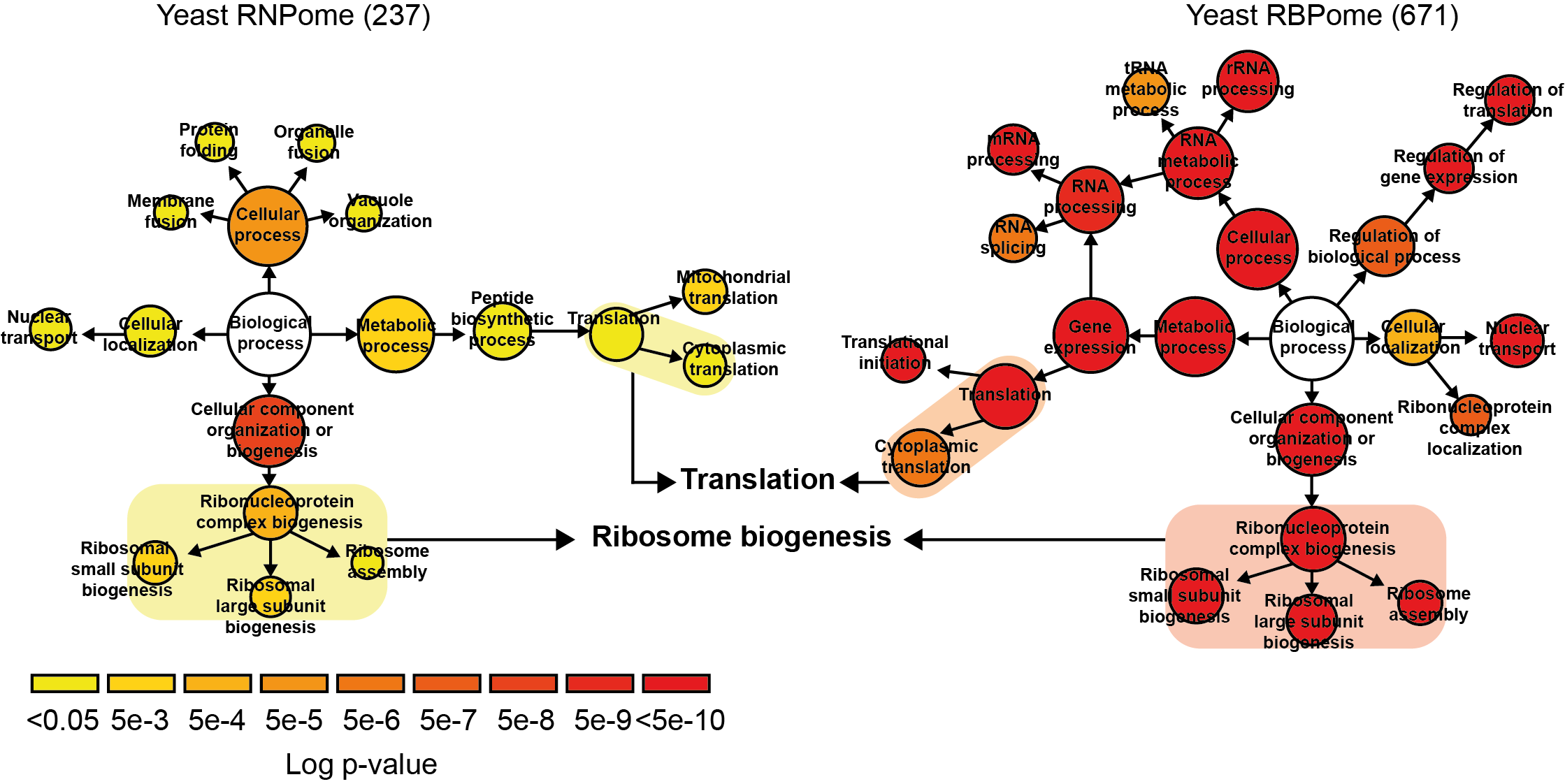

Supplement: Supplementary file 1 [file viruses-13-02190-s001.zip › Figure S3 GO comparison between the yeast RBPome and RNPome of the TSWV replicon-RGB.tif]
